# Serial CT Imaging in a Male Child at Different Ages Demonstrating the Three Types of External Occipital Protuberance

**DOI:** 10.1155/cro/8604013

**Published:** 2026-10-30

**Authors:** Kai-min Sun, Xue-feng Sun

**Affiliations:** ^1^ Department of Radiology Capital Center for Children′s Health Capital Medical University Beijing China ccmu.edu.cn

**Keywords:** case report, computed tomography, external occipital protuberance, pediatric

## Abstract

The external occipital protuberance (EOP) is a prominent bony elevation located in the posterior midline of the outer surface of the occipital bone, which can be classified into three types based on morphology. Herein, we present the case of a male child who showed all three types at different life stages. A male pediatric patient born in Beijing, China, in 2008 underwent cranial CT examinations at ages 8, 12, and 16 years for different clinical indications. Volume rendered (VR) postprocessed images revealed a dynamic evolutionary process of the EOP morphology, sequentially presenting as the smooth (Type I), wave‐peak (Type II), and spine‐like (Type III) types. We believe this is the first reported case in which a single patient fully exhibited all three types of EOP.

## 1. Introduction

The external occipital protuberance, a prominent bony elevation located in the posterior midline of the outer surface of the occipital bone, can be classified into three morphological types [1]. In our institute, we recently identified a male pediatric case in which the patient underwent cranial CT examinations at ages 8, 12, and 16 years for different clinical indications. Volume rendering (VR) of the postprocessed images demonstrated a dynamic evolutionary process of the external occipital protuberance morphology, sequentially presenting as the smooth type (Type I), the wave‐peak type (Type II), and the spine‐like type (Type III). Although prior studies have described age‐ and sex‐related differences in EOP morphology, longitudinal imaging evidence documenting its developmental progression within the same individual remains scarce. To our knowledge, such complete sequential evolution within a single individual has rarely been documented in the literature. This case report adheres to the CARE guidelines.

## 2. Case Report

While conducting a retrospective analysis of the external occipital protuberance using images from our radiology department′s picture archiving and communication system (PACS), we identified an interesting case showing evolution in this body part. This retrospective study was granted ethical approval by the Ethics Committee of Capital Center for Children′s Health, Capital Medical University (Approval No. SHERLLM2026027). The requirement for written informed consent was waived by the committee due to the noninterventional nature of the study and the anonymization of all patient data. All procedures were performed in accordance with the Declaration of Helsinki. The patient was a male child residing in Beijing, China, born in August 2008, who had undergone multiple cranial computed tomography (CT) examinations in the radiology department. In November 2016, at the age of 8 years, the boy presented to the emergency department of our hospital due to head trauma. Physical examination at that time revealed no obvious abnormalities. CT images showed no signs of significant craniocerebral or soft tissue injury, with no obvious structural abnormalities in the brain parenchyma; the external occipital protuberance was classified as the smooth type (Type I), appearing arc‐shaped (see Figure 1A,B). In August 2020, at the age of 12 years, the boy presented to the neurosurgery department of our hospital owing to a small subcutaneous mass in the occipital region. Physical examination revealed a small midline mass in the occipital region, with no obvious surface abnormalities and no significant tenderness on palpation. CT images at this time demonstrated that the external occipital protuberance was of the wave‐peak type (Type II), presenting a mountain‐peak shape (see Figure 1C,D). In the diagnostic report, we provided an objective description stating that a pointed bony protrusion was observed in the midline of the occipital bone, with clear borders, measuring approximately 4 × 5 × 4 mm, with no bone destruction, no periosteal reaction, and no mass formation observed in the surrounding soft tissues. In September 2024, at the age of 16 years, the boy presented to the emergency department of our hospital after experiencing dizziness and headache following trauma. Physical examination revealed no obvious abnormalities. His CT images showed no signs of significant craniocerebral or soft tissue injury, and no obvious structural abnormalities in the brain parenchyma. However, at this time, the external occipital protuberance was of the spine‐like type (Type III), appearing beak‐shaped and spine‐like (see Figure 1E,F). The clinical course is presented chronologically in Table 1.

**Figure 1:**
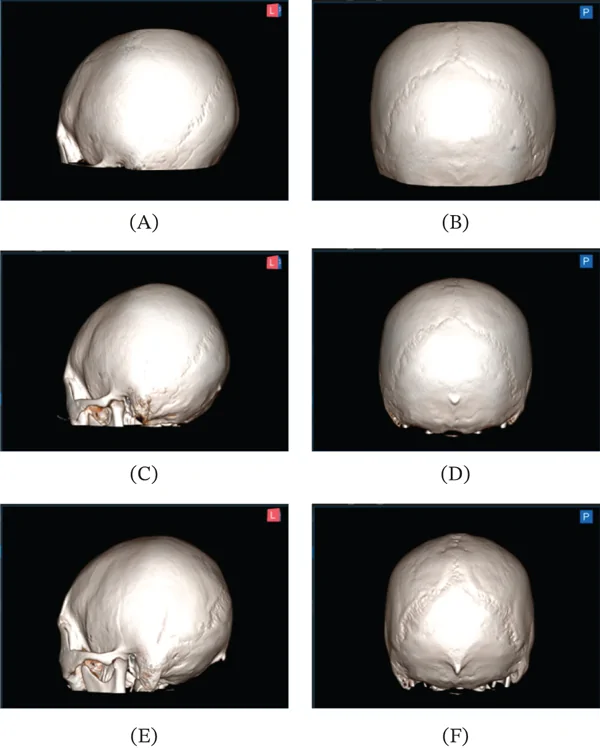
VR images showing the three different types of external occipital protuberance. (A,B) At 8 years of age, the boy′s external occipital protuberance was Type I (smooth type). (C,D) At 12 years of age, the boy′s external occipital protuberance was Type II (wave‐peak type). (E–F) At 16 years of age, the boy′s external occipital protuberance was Type III (spine‐like type).

**Table 1:** Clinical timeline.

Time point	Age	Clinical event	EOP types
November, 2016	8 years	Presented to the emergency department due to head trauma	Type I
August, 2020	12 years	Presented to the neurosurgery department owing to a small subcutaneous mass in the occipital region	Type II
September, 2024	16 years	Presented to the emergency department of our hospital after experiencing dizziness and headache following trauma	Type III

## 3. Discussion

The overall thickness of the occipital bone in children is consistently greater in the midline and paramedian regions than in more lateral areas [2]. However, as children age, the proportion of Type II external occipital protuberance increases significantly, whereas Type III external occipital protuberance generally appears in late adolescence [3]. In young adult populations, Type I external occipital protuberance is more common in females, whereas Types II and III are more common in males [4]. The external occipital protuberance represents a type of bony spur, and chronic tensile forces from the nuchal ligament induce reactive osteogenesis at the EOP attachment site [5, 6]. Atadağ et al. argued that biomechanical and immunologic factors may constitute the main determinants of EOP formation in children [3]. In addition, Cheng et al. reported that adult subjects with EOP exhibited more severe forward head posture and larger facial configuration [7]. The external occipital protuberance is mostly asymptomatic, but may cause occipital pain or shoulder pain [8, 9], and can also result in fractures [10]. Recognizing and understanding the classification and evolutionary process of the external occipital protuberance in both children and adults offers substantial benefits for research in related fields such as anatomy, forensic medicine, and radiology. This study has several limitations. As this is a single case report, the findings cannot be generalized to all pediatric and adolescent populations, and larger longitudinal studies are needed to validate our observations.

## 4. Conclusion

Serial CT Imaging can reveal a dynamic evolutionary process of EOP morphology in children.

## Author Contributions

Kai‐min Sun conceived and designed the study, analyzed the data, and drafted the manuscript. Xue‐feng Sun supervised the study and approved the final version of the manuscript.

## Funding

This study was supported by the Capital Center for Children′s Health, Capital Medical University (FZYJ‐2027‐20).

## Disclosure

All authors have read and agreed to the published version of the manuscript.

## Consent

No written consent has been obtained from the patients, as there is no patient identifiable data included in this case report.

## Conflicts of Interest

The authors declare no conflicts of interest.

## Data Availability

The data that support the findings of this study are available on request from the corresponding author. The data are not publicly available due to privacy or ethical restrictions.

## References

[1] Gülekon I. N. and Turgut H. B. , The External Occipital Protuberance: Can It Be Used as a Criterion in the Determination of Sex?, Journal of Forensic Sciences. (2003) 48, no. 3, 513–516, 10.1520/JFS2002183, 12762519. 12762519

[2] Wang Y. L. , Xu H. M. , Wang X. Y. , Li Y. , Shen Z. H. , Zhou K. L. , Zhou F. , Jin H. M. , Chen J. X. , and Kong Q. Y. , A Computed Tomographic Morphometric Study of the Pediatric Occipital Bone Thickness, Spine. (2015) 40, no. 20, 1564–1571, 10.1097/BRS.0000000000001091, 26731701. 26731701

[3] Atadağ A. , Cihan Ö. F. , Artaş A. , Aşantoğrol F. , Sönmezışık S. , Çetin C. Ş. , Şencan Z. , and Beger O. , Age-Related Alterations in the Shape of the External Occipital Protuberance in Children, Surgical and Radiologic Anatomy. (2025) 47, no. 1, 10.1007/s00276-025-03694-w, 40760059. 40760059

[4] Jacques T. , Jaouen A. , Kuchcinski G. , Badr S. , Demondion X. , and Cotten A. , Enlarged External Occipital Protuberance in Young French Individuals′ Head CT: Stability in Prevalence, Size and Type Between 2011 and 2019, Scientific Reports. (2020) 10, no. 1, 10.1038/s41598-020-63554-y, 32300156. PMC7162866 32300156

[5] Benjamin M. , Toumi H. , Ralphs J. R. , Bydder G. , Best T. M. , and Milz S. , Where Tendons and Ligaments Meet Bone: Attachment Sites (′Entheses′) in Relation to Exercise and/or Mechanical Load, Journal of Anatomy. (2006) 208, no. 4, 471–490, 10.1111/j.1469-7580.2006.00540.x, 16637873. PMC2100202 16637873

[6] Gong Z. , Sun H. , Li D. , Lu X. , Liu S. , Wang X. , Xia X. , Lyu F. , Jiang J. , Zou F. , Wang H. , and Ma X. , Gender-Specific Patterns of External Occipital Protuberance Hyperplasia: Associations With Nuchal Ligament Ossification and Cervical Sagittal Imbalance in Myelopathy Patients, Asian Spine Journal. (2026) 20, no. 1, 10–19, 10.31616/asj.2025.0109, 40858317. PMC12960429 40858317

[7] Cheng Q. , Xiao C. , Lau Rui Han S. , Hu S. , Wu X. , Dong Y. , Xiong X. , and Fang S. , The Association of Occipital Spur With Craniocervical Posture and Craniofacial Morphology, Journal of Pain Research. (2025) 18, no. 18, 179–187, 10.2147/JPR.S488467. PMC11731010 39811251

[8] Satyarthee G. D. , External Occipital Protuberance Projecting as Downward Curved Horn Presenting With Intractable Occipital Pain: Report of a First Case, Journal of Pediatric Neurosciences. (2019) 14, no. 3, 173–174, 10.4103/jpn.JPN_94_18, 31649782. PMC6798274 31649782

[9] Aydn M. E. and Atik A. , Can External Occipital Protrusion Be the Cause of Shoulder Pain?, Journal of the Anatomical Society of India. (2025) 74, no. 4, 363–365, 10.4103/jasi.jasi_48_25.

[10] Sattur M. , Korson C. , Henderson F. , and Kalhorn S. , Presentation and Management of Traumatic Occipital Spur Fracture, American Journal of Emergency Medicine. (2019) 37, 1005.e1–1005.e2, 10.1016/j.ajem.2019.01.043, 30733104. 30733104

